# Influences of Different CAD/CAM Ceramic Compositions and Thicknesses on the Mechanical Properties of Ceramic Restorations: An In Vitro Study

**DOI:** 10.3390/ma16020646

**Published:** 2023-01-09

**Authors:** Passent Ellakany, Marwa Madi, Nourhan M. Aly, Turki Alshehri, Shahad T. Alameer, Fahad A. Al-Harbi

**Affiliations:** 1 Department of Substitutive Dental Sciences, College of Dentistry, Imam Abdulrahman Bin Faisal University, Dammam 32210, Saudi Arabia; 2 Department of Preventive Dental Sciences, College of Dentistry, Imam Abdulrahman Bin Faisal University, Dammam 32210, Saudi Arabia; 3 Department of Pediatric Dentistry and Dental Public Health, Faculty of Dentistry, Alexandria University, Alexandria 21527, Egypt; 4 College of Dentistry, Imam Abdulrahman Bin Faisal University, Dammam 32210, Saudi Arabia

**Keywords:** CAD/CAM, ceramics, roughness, hardness, toothbrushing, thickness

## Abstract

The aim of this study was to assess the influences of different CAD/CAM ceramic compositions and thicknesses on the surface roughness and hardness of ceramic restorations. Four different ceramics were used in the current study: lithium disilicate (LD), leucite reinforced (LE), advanced lithium disilicate (ALD), and zirconia-reinforced lithium silicate (ZLS). Each group included 30 specimens subdivided into three different ceramic thicknesses (0.5, 1 and 1.5 mm thicknesses). The microhardness was measured for all the specimens using a microhardness testing machine, while the surface roughness was measured using a non-contact optical profilometer at three intervals (before toothbrushing and after toothbrushing, with and without toothpaste). Three-way and two-way ANOVA were used to determine the factors influencing the surface roughness and microhardness. There was a significant difference in the surface roughness between the studied groups for all the thicknesses. The findings showed that ALD had the lowest surface roughness, while ZLS showed the highest surface roughness. Moreover, ALD, followed by ZLS, had the highest hardness, while LD and LE had the lowest hardness values. Regarding the thicknesses, both the 0.5 and 1 mm ceramic thicknesses showed a significantly lower surface roughness than the 1.5 mm thickness, while the 1.5 mm thickness showed a significantly higher microhardness than the 0.5 mm thickness. The surface roughness and hardness were significantly affected by the ceramic composition and type of filler. It is recommended to use 1.5 mm-thick ceramic materials for the fabrication of definitive full-coverage ceramic restorations, while veneers require 0.5 mm-thick materials. ALD is a promising CAD/CAM material that can be used for the fabrication of restorations with a proper strength in both anterior and posterior regions.

## 1. Introduction

Computer-aided design and computer-aided manufacturing (CAD/CAM) methods enable the fabrication of machined restorations with acceptable shade, strength, and marginal adaptation at an affordable cost [1,2]. Monolithic millable blocks provide the definitive restoration of single materials, unlike the multiple layering of the ceramic restoration. This results in higher material qualities, such as a high fracture toughness, hardness, acceptable esthetics, and minimal adjustments in the oral cavity, limiting the extent of ceramic fracture or chipping [3,4]. The improvement in the clinical performance of monolithic restorations may be attributed to the lack of differences in their flexural strength, modulus of elasticity, and thermal expansion coefficient, unlike multilayered ceramic restorations that are composed of several ceramic layers of different compositions [5,6]. Sulaiman et al. [7] showed that dental veneers fabricated from monolithic lithium disilicate (LD) CAD/CAM blocks had a higher strength, hardness, and better esthetics when compared to multilayered ceramic blocks. Among the available CAD/CAM ceramics, lithium disilicate (LD), leucite-reinforced (LE) glass ceramics, and zirconia have proven to be the most favorable ceramic materials used in the oral cavity [8,9,10]. LD ceramic is considered the material of choice for the fabrication of posterior and anterior restorations because of its superior mechanical and esthetic properties [11,12]. Previous studies reported that LD ceramics showed comparable 5-year survival rates to those of zirconia when used for the fabrication of posterior crowns and fixed partial dentures [13,14].

Recently, the addition of reinforcing dental ceramic components to a resin matrix has shown improvements in the biocompatibility and physical properties of restorative materials, in addition to providing an even distribution of the masticatory forces [15]. One of these commonly used materials is BioHPP^®^, which is composed of partially crystalline polyether ether ketone (PEEK) reinforced with ceramic, providing an acceptable balance between the elasticity and rigidity properties, with high esthetic features [16]. Various fixed prostheses have been developed using this material, including single crowns, short span fixed partial dentures (FPDs), and resin-bonded FPDs [17].

Resin matrix ceramics are a new ceramic material that combine the advantages of ceramics and polymers [18]. They share many properties with ceramic materials, such as their esthetics, strength, adhesion, and wear resistance, and similar to dentin, they have a low elastic modulus [19]. The greatest advantage of these ceramics is their superior sidewall retention and excellent stability when used for crown repair bodies, inlays, and onlays [20].

Another type that has also been developed is polymer-infiltrated ceramic, which is termed a hybrid ceramic. This material can be conditioned in the same manner, as either an indirect composite or an etchable ceramic [21]. Due to their good mechanical and biological properties [22], these products are suitable materials for the fabrication of crown, inlay, and onlay restorations [23].

Zirconia-reinforced lithium silicate ceramic (ZLS) was introduced to the market with the advantage of being compatible with CAD/CAM technologies [24]. It was suggested that this structural composition offers satisfactory optical properties and an enhanced mechanical behavior compared to other glass ceramics [25]. Since ZLS is highly transparent and has strong biaxial flexural properties, it has been used for implant-supported restorations and tooth esthetic restorations, including veneers [17,26].

Recently, a new glass ceramic material named advanced lithium disilicate glass ceramic (ALD; CEREC Tessera) was developed [27]. ALD is composed of lithium aluminum silicate crystals known as virgilite [27,28]. According to manufacturer specifications, virgilite crystals provide a higher strength and esthetic properties, rendering them the material of choice for the fabrication of inlays, onlays, crowns, and veneers [28,29].

Another factor to be considered in the fabrication of definitive restorations is the minimum ceramic thickness that can enable conservative tooth preparation, maintain pulp vitality, and provide the retention and resistance of the final restoration [30]. A ceramic thickness in the range of 0.5–1 mm can be used for the fabrication of veneers [30], while a thickness of at least 1.5 mm has shown to be appropriate for the fabrication of full-coverage CAD/CAM ceramic restorations [12,31]. Previous studies demonstrated an acceptable fracture resistance of monolithic zirconia crowns with a 0.5 mm thickness fabricated on the posterior molars [32,33]. However, further research using other types of ceramics, such as ZLS, ALD, LD, and LE, is still required.

Surface roughness is one of the factors that influence the clinical survival of ceramics, since it might result in the wear and abrasion of the ceramics, crack initiation that can lead to fracture and staining, a loss of translucency, and bacterial colonization [34,35,36,37]. Previous studies have reported that the surface roughness threshold should be less than 0.2 µm in order to avoid plaque accumulation and bacterial adhesion on prosthetic materials [38,39]. Multiple factors have been shown to have a direct influence on the surface roughness of ceramic materials, including the type of ceramic, thickness, polishing methods, polishing materials, routine toothbrushing, and the toothpaste used, as some toothpastes include abrasive particles designed to enhance cleaning [34,35,37]. Moreover, the hardness of the ceramic can be influenced by the variable ceramic thicknesses used in the oral cavity to withstand masticatory forces without compromising the tooth structure or esthetics [34].

Several studies have assessed the effects of toothbrushing on the surface roughness of CAD/CAM dental ceramics [37,40,41]. Nevertheless, data on the micro-hardness and surface roughness of the ALD are still limited and require further exploration. Therefore, the aim of the current study was to assess the influences of different CAD/CAM ceramic compositions and thicknesses on the surface roughness and hardness of definitive ceramic restorations. The null hypothesis was that there would be (1) no difference in the surface roughness and hardness of the different ceramic compositions, and (2) the surface roughness and hardness of each ceramic material tested, with the variable thicknesses, would show comparable results.

## 2. Materials and Methods

### 2.1. Sample Grouping

**Figure 1:**
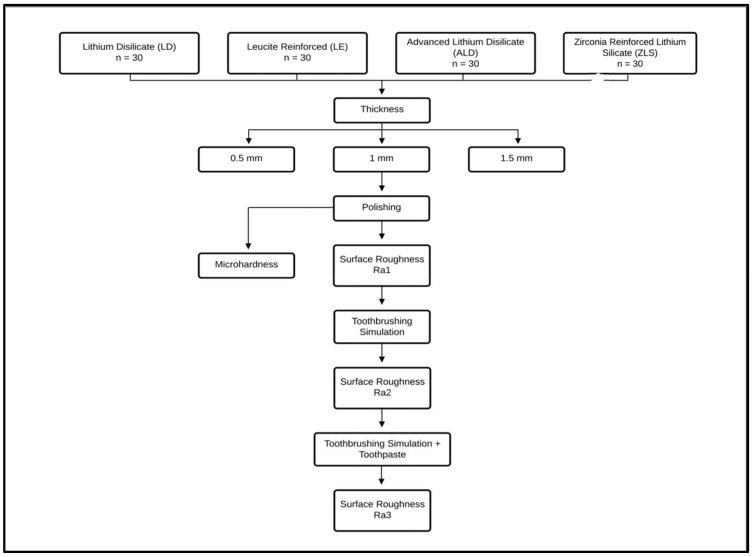
Diagrammatic representation of the study design and study groups.

Four different types of dental CAD/CAM ceramics of shade A1 with a low translucency were used in the current study, as follows: lithium disilicate (IPS Emax CAD, Ivoclar Vivadent, Schaan, Liechtenstein; LD), leucite reinforced (IPS Empress CAD, Ivoclar Vivadent, Schaan, Liechtenstein; LE), advanced lithium disilicate (Cerec Tessera, Sirona Dentsply, Milford, DE, USA; ALD), and zirconia-reinforced lithium silicate (Celtra Duo, Sirona Dentsply, Milford, DE, USA; ZLS). Each group of ceramics included 30 ceramic specimens subdivided into three different ceramic thicknesses (n = 10 per subgroup) reflecting the most common ceramic thicknesses (0.5 mm, 1 mm and 1.5 mm thicknesses), with a total sample size of 120 specimens. The sample size was estimated using G*Power (Version 3.1.9.4) with an 80% study power and 5% alpha error to detect an effect size = 0.59 [42]. The minimum sample size was calculated to be 9 per group, increased to 10 in order to compensate for laboratory processing errors. The total sample size = the number of subgroups × number per subgroup = 12 × 10 = 120), as shown in Figure 1.

### 2.2. Specimens’ Preparation

The specimens were sectioned using a precision cutting machine (Isomet 5000, Buehler, Lake Bluff, IL, USA) with copious cooling water into the thicknesses of 0.5, 1, and 1.5 mm, with the same dimensions of the block, according to the International Organization of Standards (ISO standards 6872:2015) [43]. The specimens were polished using 500-grit silicon carbide discs for 60 s at a speed of 200 rpm with a water-cooling system, using a polishing machine (MetaServ 250 Grinder-Polisher with Vector Power Head, Buehler, IL, USA). Following this step, the specimens were crystallized according to the manufacturer’s instructions in a ceramic furnace (Programat EP5010, Ivoclar Vivadent, Schann, Liechtenstein). After crystallization, the specimens were polished with carbide discs of different grits (400 and 600 grits) in a wet environment for 60 s at a 200 rpm speed, using the same polishing machine mentioned previously. The dimensions of all the specimens were evaluated using a digital caliper (Mitutoyo Corp, Kawasaki, Japan) to assure that the dimensions were within a 0.05 mm thickness.

### 2.3. Microhardness Measurements

The surface microhardness was measured for all the specimens using a microhardness testing machine (MicroMet 6040, Buehler LTD., Lake Bluff, IL, USA) according to the ASTM C1327-15 standard [43]. A load of 50 gms was applied for 20 s to three different regions of each specimen through the use of Vickers hardness indenter [44].

### 2.4. Surface Roughness Measurements (Ra)

The surface roughness of the ceramic specimens was measured using a non-contact optical profilometer (Contour Gt-K 3D optical profiler, Bruker Nano GmbH, Berlin, Germany) after the polishing procedure (Ra1) was accomplished. Three readings were detected for each specimen on different regions at a speed of 0.5 mm per second, the cutoff was set to 0.8 mm, and the mean of these readings was calculated.

### 2.5. Toothbrushing Simulation Procedure

The specimens were placed in a putty index holder and subjected to a toothbrushing procedure (T1) using the toothbrushing simulation machine (model ZM-3.8; SD Mechatronik GmbH, Feldkirchen-Westerham, Germany) for 40,000 strokes, where 75 strokes were applied for each 60 s using a toothbrush (Colgate Twister, Colgate-Palmolive, São Paulo, SP, Brazil), with a total duration of 4 h and 44 min and a load of 0.2 Newtons [45]. Surface roughness measurements (Ra2) were repeated for all the specimens after the brushing procedure. The same brushing procedure was repeated using toothpaste (T2; Colgate, Colgate-Palmolive Company, Sao Paulo, SP, Brazil) with a low abrasive effect (RDA = 63), where 250 g of paste was dissolved in 1 L of distilled water [46,47,48], according to ISO 11609:2010 [49]. Then, all the specimens were cleaned with distilled water and dried to detect the surface roughness for the third time (Ra3).

### 2.6. Statistical Analysis

The normality was verified using descriptive statistics, plots (Q-Q plots and histograms), and normality tests. The means and standard deviations were calculated for all the variables. Comparisons between the studied groups were performed using one-way ANOVA and Kruskal–Wallis tests, according to the variables’ normality. Three-way ANOVA was used to determine the association between the surface roughness and study material, thickness, and toothbrushing, while two-way ANOVA was used to assess the association between the hardness and both the study material and thickness. Adjusted means, standard errors, 95% confidence intervals, and adjusted R2 were calculated. The significance value was inferred at *p* < 0.05. The data were analyzed using IBM SPSS for Windows (Version 23.0).

## 3. Results

**Figure 2:**
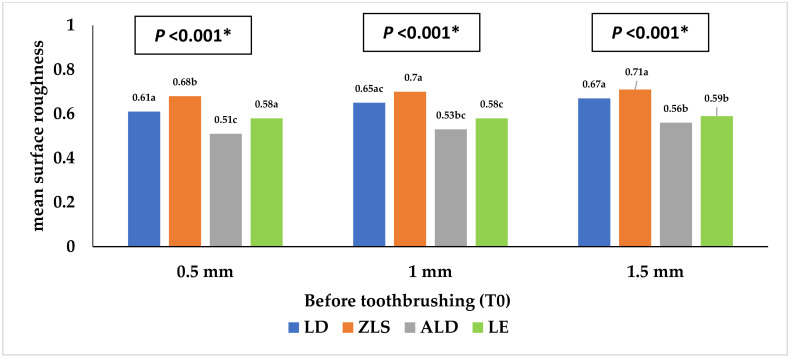
Mean surface roughness (Ra1) of the four study groups before toothbrushing. * Statistically significant at *p* value < 0.05. **a**, **b**, **c**: Different lowercase letters denote statistically significant differences between the four groups using the Bonferroni-adjusted significance level.

**Figure 3:**
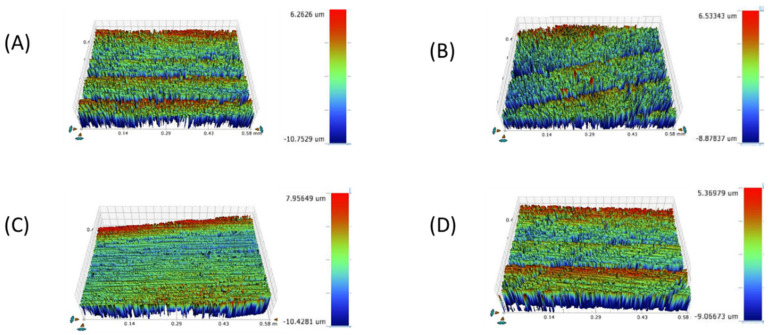
Surface roughness micrograph of tested CAD/CAM ceramics after polishing (Ra1). (**A**) LD, (**B**) ZLS, (**C**) ALD, (**D**) LE.

**Figure 4:**
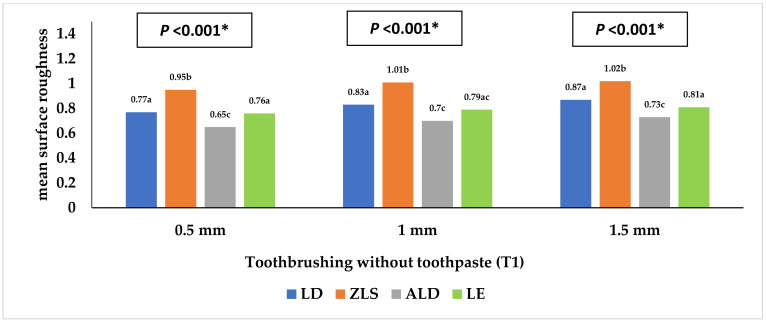
Mean surface roughness (Ra2) of the four study groups after toothbrushing without toothpaste. * Statistically significant at *p* value < 0.05. **a**, **b**, **c**: Different lowercase letters denote statistically significant differences between the four groups using the Bonferroni-adjusted significance level.

**Figure 5:**
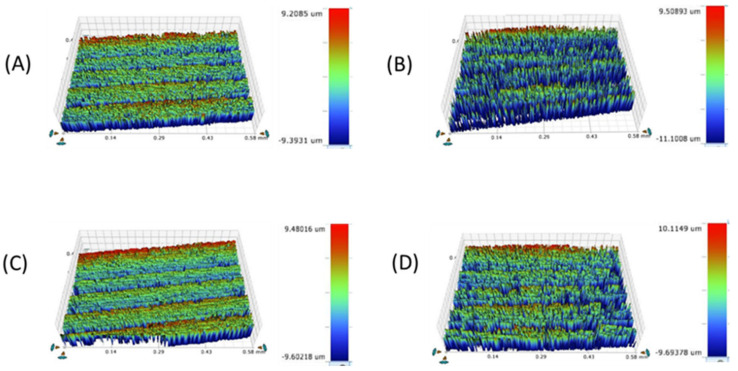
Surface roughness micrograph of the tested CAD/CAM ceramics after brushing without toothpaste (Ra2). (**A**) LD, (**B**) ZLS, (**C**) ALD, (**D**) LE.

**Figure 6:**
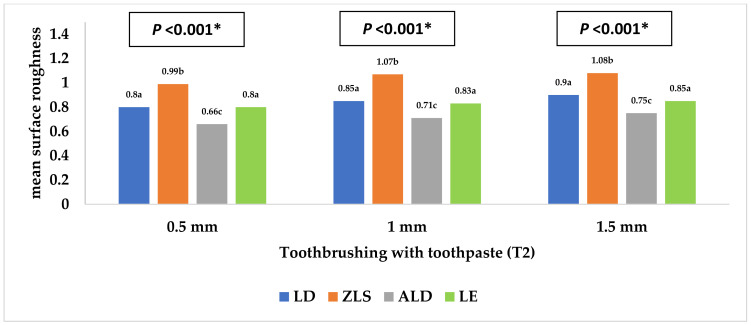
Mean surface roughness (Ra3) of the four study groups after toothbrushing with toothpaste. * Statistically significant at *p* value < 0.05. **a**, **b**, **c**: Different lowercase letters denote statistically significant differences between the four groups using the Bonferroni-adjusted significance level.

**Figure 7:**
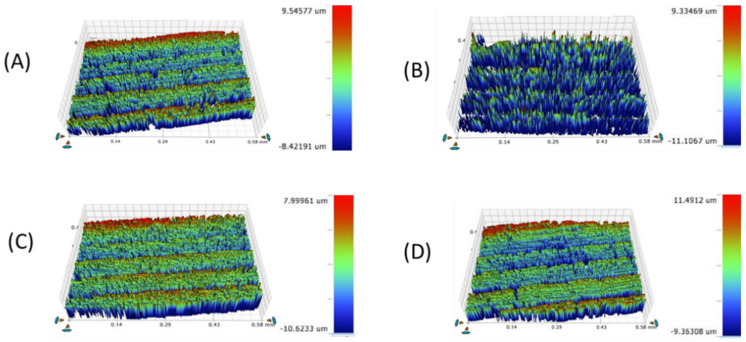
Surface roughness micrograph of the tested CAD/CAM ceramics after toothbrushing with toothpaste (Ra3). (**A**) LD, (**B**) ZLS, (**C**) ALD, (**D**) LE.

Table 1 and Figure 2, Figure 3, Figure 4, Figure 5, Figure 6 and Figure 7 represent the surface roughness of the four study groups at different thicknesses before and after brushing. There was a significant difference in the surface roughness between the studied groups at all the thicknesses (*p <* 0.001). The ZLS group showed the highest mean (SD) surface roughness among all the groups at all the thicknesses, followed by the LD and LE groups, while ALD showed the lowest mean (SD) values. When comparing the roughness of the different thicknesses for the same material, it was noted that the mean (SD) values increased with the increasing thickness in all the groups. Moreover, the values increased after toothbrushing with and without toothpaste.

Table 2 shows the differences in the surface roughness of the studied groups before and after toothbrushing. At the 0.5 thickness, the difference in roughness after toothbrushing with toothpaste, compared to before, was significantly higher in the LE group than the LD group (mean (SD) = 0.31 (0.10) compared to 0.19 (0.06)), with no significant difference detected between the other groups. At the 1 mm thickness, the difference in roughness after toothbrushing with and without toothpaste, compared to before, was significantly different between the LD and both the ALD and LE groups, with no other significant differences detected. Thus, the mean (SD) difference in the surface roughness was comparable for both LD and ZLS and also for the ALD and LE groups, respectively.

**Figure 8:**
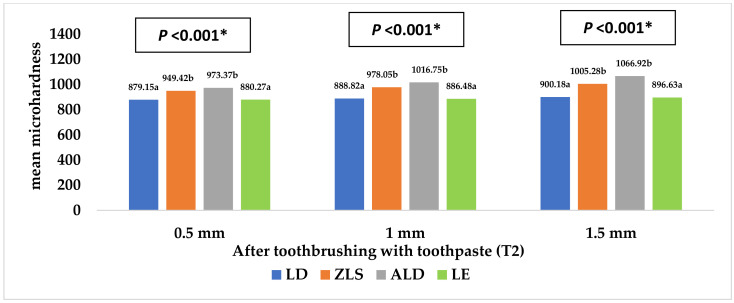
Mean microhardness of the four study groups after toothbrushing with toothpaste. * Statistically significant at *p* value < 0.05. **a**, **b**: Different lowercase letters denote statistically significant differences between the four groups using the Bonferroni-adjusted significance level.

**Figure 9:**
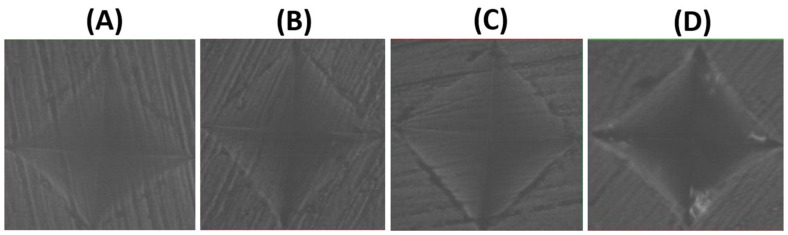
Microhardness topography (×40) of the tested CAD/CAM ceramics: (**A**) LD, (**B**) ZLS, (**C**) ALD, (**D**) LE.

Table 3 and Figure 8 and Figure 9 represent the surface microhardness of the studied materials at different thicknesses. There was a significant difference between LD and both the ZLS and ALD groups at all the thicknesses (*p <* 0.001). The highest mean (SD) microhardness values were reported in the ALD group, while the lowest were reported in the LE group. Comparing the different specimen thicknesses in the same group, the 1.5 mm thickness showed a significantly higher hardness than the 0.5 mm thickness in both the ZLS and ALD groups (*p* = 0.008), with no significant differences detected between the other groups (*p* > 0.05).

Table 4 shows the different factors associated with the surface roughness. As for the study material, there were significant differences between the four studied groups (*p <* 0.001), with the highest values reported in the ZLS group, followed by the LD and LE groups, while the lowest value was reported in the ALD group (adjusted means = 0.91, 0.77, 0.73, and 0.64 µm, respectively). Regarding the specimen thickness, the surface roughness increased significantly with the increasing specimen thickness (adjusted means = 0.73, 0.77, and 0.80 µm for 0.5, 1, and 1.5 mm thicknesses, respectively, *p <* 0.001). Moreover, the surface roughness before toothbrushing was significantly lower than that after toothbrushing (with and without toothpaste use) (*p <* 0.001).

Table 5 shows the different factors associated with hardness. Regarding the study material, both LD and LE had a significantly lower surface roughness than the ZLS and ALD groups (adjusted means = 889.38, 887.79, 977.58, and 1019.01 mm for LD, LE, ZLS, and ALD, respectively, *p <* 0.001). As for the thickness, the hardness was significantly higher in the case of the 1.5 mm than the 0.5 mm thickness (adjusted means = 967.25 mm and 920.55 mm for 1.5 and 0.5 mm, respectively, *p <* 0.001).

## 4. Discussion

The findings of the present study showed that ALD had the lowest surface roughness, while ZLS showed the highest surface roughness among the four tested ceramics. When assessing the hardness, we found that ALD, followed by ZLS, had the highest hardness, while LD and LE had the lowest hardness values. Regarding the ceramic thicknesses, both the 0.5 mm and 1 mm ceramic thicknesses showed a significantly lower surface roughness than the 1.5 mm thickness. Therefore, both hypotheses were rejected.

When comparing the levels of surface roughness of the tested materials, ZLS exhibited the highest surface roughness, while ALD had the lowest surface roughness, with significant differences between all four ceramic materials. These differences might be related to the variations in the ceramic composition and size of the fillers. ZLS is composed of zirconia-reinforced lithium silicate particles, while the ALD composition includes lithium disilicate and virgilite, a lithium aluminum silicate. This observation is in agreement with Matzinger et al.’s [50] findings, which showed a higher surface roughness in ZLS specimens when compared to LD, resin-infiltrated ceramics, and composite specimens. The difference was explained by the presence of zirconium dioxide (ZrO2) particles in ZLS, which act as abrasive agents that result in the deterioration of the surface roughness [50,51,52]. Moreover, another study reported minimal wear in the case of ALD when compared to LD [27]. This high wear resistance enables the maintenance of the smooth surface of ALD and limits the wear tendency of the opposing dentition. ALD is an innovative ceramic material that requires further study in order to compare its properties to the various available CAD/CAM ceramics.

Our results showed that LE exhibited a significantly smoother surface than LD, which is in agreement with the findings documented in the literature [53,54]. However, when assessing Ra in the three stages of the current study, LE and LD exhibited comparable Ra readings, with smoother surfaces in the case of LE. This might be explained by the differences in composition between LE and LD, since LE includes leucite particles that are easily polished compared to the lithium disilicate particles of LD. Thus, LD will require multiple polishing procedures and various polishing agents to achieve the same surface topography as LE [54]. Another study is in agreement with the Ra findings of the current study, where the authors showed a significant increase in the surface roughness of LD and ZLS compared to LE after polishing the specimens [35]. The surface roughness after toothbrushing with toothpaste (Ra3) was comparable to that following the toothbrushing process without toothpaste (Ra2).

The use of non-abrasive toothpaste in the toothbrushing procedure, similar to previous studies, might be the main factor contributing to the similarity of the Ra readings with and without toothpaste (Ra2 and Ra3), as the abrasive index of the used toothpaste is low [48,55]. Similarly, Flurry et al. [56] reported significant reductions in the surface roughness of their tested ceramics using a toothpaste of reduced abrasive value (70 RDA). Despite this, the toothbrushing procedure itself caused a significant increase in the surface roughness because of the abrasive motion of the brushing process, simulating the environment inside the patient’s oral cavity. Toothpaste and toothbrush bristles might cause the abrasion of the ceramic surfaces by creating minor superficial grooves, and this observation is consistent with other studies [37,40,41]. The Ra readings of all the tested materials in all the subgroups of ceramic thicknesses exceeded 0.2 μm, which is considered to be the plaque accumulation threshold and, thus, roughness can be easily detected by the tongue and can lead to irritation and bacterial colonization [45,48,54]. This might be due to the lack of application of a diamond polishing paste following the conventional polishing procedure, used to achieve a smoother surface [35,57]. Thus, the application of a diamond polishing paste should be considered in future studies to compare its effect with the application of a glazed material.

When evaluating the effects of the ceramic thickness on the hardness of the ceramic materials, the 1.5 mm thickness showed the highest hardness, followed by the 1 mm and 0.5 mm thicknesses. Therefore, this finding supports the benefit of limiting the use of a 0.5 mm ceramic thickness in the fabrication of dental veneers for esthetic purposes only, while the use of a 1 mm thickness can be extended to the fabrication of inlays and onlays. The 1.5 mm thickness can be considered the optimum thickness for the fabrication of full-coverage restorations subjected to heavy masticatory loads [12,31].

When examining the impact of the ceramic composition on the hardness property, ALD exhibited the highest hardness, followed by ZLS and LD, respectively, while LE was the weakest material tested. ZLS includes zirconia fillers that incorporated at the level of 10% of the weight of the total composition, and this might explain its higher hardness compared to LE and LD [58]. Colombo et al. [59] reported that ZLS had the highest hardness when compared to hybrid ceramics and resin nano-ceramics. However, Corade et al. [43] stated that LD showed a higher hardness than ZLS, which is inconsistent with the current findings. This might be related to the different methodology, ceramic specimen dimensions, absence of polishing procedures, and the use of non-crystallized ZLS, which differed from that used in the current study.

In comparison to the other lithium disilicate glass ceramics, ALD ceramics showed higher hardness levels that could make them an attractive clinical choice for restorations in high-stress-bearing areas. Moreover, the chairside processing of ALD ceramics is simpler, quicker, and more practical than that of zirconia ceramics [60].

ALD is an innovative CAD/CAM ceramic material that requires further study in order to assess its biological and optical properties. In addition, by comparing the mechanical properties of short- and long-span FPDs fabricated from the tested ceramics, we can simulate the restorations fabricated inside the patient’s oral cavity. Future studies are recommended to evaluate the color stability of different ceramic thicknesses in relation to various staining solutions, in addition to implementing clinical studies designed to replicate the oral environment’s features, including the temperature, pH, masticatory forces, and the presence of saliva.

## 5. Conclusions

ALD exhibited the highest hardness and surface smoothness in comparison to the other tested ceramics. Differences in the hardness and surface roughness were significantly affected by the ceramic composition and type of filler included in the ceramics. The optimum thickness of the ceramic material to be used for the fabrication of definitive full-coverage ceramic restoration is 1.5 mm, while 0.5 mm should be restricted to the fabrication of ceramic veneers. ALD is a promising CAD/CAM ceramic material that can be used for the fabrication of restorations with a proper translucency and strength in both anterior and posterior regions.

## Figures and Tables

**Table 1:** Surface roughness of the four study groups at different thicknesses and different timepoints.

Timepoints	Thickness	LD (n = 10)	ZLS (n = 10)	ALD (n = 10)	LE (n = 10)	*p* Value
		Mean (SD)				
**T1**	**0.5 mm**	0.61 (0.04) **a, A**	0.68 (0.05) **b**	0.51 (0.05) **c**	0.58 (0.04) **a**	<0.001 *
	**1 mm**	0.65 (0.06) **ac, AB**	0.70 (0.05) **a**	0.53 (0.07) **bc**	0.58 (0.05) **c**	<0.001 *
	**1.5 mm**	0.67 (0.05) **a, B**	0.71 (0.07) **a**	0.56 (0.06) **b**	0.59 (0.04) **b**	<0.001 *
	***p*** **value**	0.04 *	0.41	0.22	0.72	
**T2**	**0.5 mm**	0.77 (0.06) **a, A**	0.95 (0.06) **b, A**	0.65 (0.08) **c**	0.76 (0.08) **a**	<0.001 *
	**1 mm**	0.83 (0.07) **a, AB**	1.01 (0.04) **b, B**	0.70 (0.09) **c**	0.79 (0.08) **ac**	<0.001 *
	**1.5 mm**	0.87 (0.04) **a, B**	1.02 (0.06) **b, B**	0.73 (0.08) **c**	0.81 (0.07) **a**	<0.001 *
	***p*** **value**	0.003 *	0.007 *	0.12	0.47	
**T3**	**0.5 mm**	0.80 (0.07) **a, A**	0.99 (0.09) **b**	0.66 (0.09) **c**	0.80 (0.06) **a**	<0.001 *
	**1 mm**	0.85 (0.06) **a, AB**	1.07 (0.09) **b**	0.71 (0.07) **c**	0.83 (0.06) **a**	<0.001 *
	**1.5 mm**	0.90 (0.06) **a, B**	1.08 (0.09) **b**	0.75 (0.07) **c**	0.85 (0.07) **a**	<0.001 *
	***p*** **value**	0.003 *	0.08	0.06	0.15	

LD: IPS Emax, ZLS: Celtra Duo, ALD: Cerec Tessera, LE: IPS Empress. T1: Before toothbrushing. T2: After toothbrushing (without toothpaste). T3: After toothbrushing (with toothpaste). One-way ANOVA was used. * Statistically significant at *p* value < 0.05. **a**, **b**, **c**: Different lowercase letters denote statistically significant differences between the four groups using the Bonferroni-adjusted significance level. **A**, **B**: Different uppercase letters denote statistically significant differences between the different thicknesses in the same group using the Bonferroni-adjusted significance level.

**Table 2:** Difference in the surface roughness of the four study groups at different thicknesses.

Ceramic Thicknesses	Thickness	LD (n = 10)	ZLS (n = 10)	ALD (n = 10)	LE (n = 10)	*p* Value
		Mean (SD)				
**0.5 mm**	**T2-T1**	0.16 (0.10)	0.18 (0.11)	0.21 (0.06)	0.27 (0.08)	0.07
	**T3-T1**	0.19 (0.06) **a**	0.20 (0.07) **a, b**	0.23 (0.08) **a, b**	0.31 (0.10) **b**	0.02 *
	**T3-T2**	0.03 (0.10)	0.02 (0.10)	0.03 (0.07)	0.04 (0.13)	0.99
**1 mm**	**T2-T1**	0.32 (0.06) **a**	0.31 (0.08) **a**	0.14 (0.08) **b**	0.17 (0.10) **b**	<0.001 *
	**T3-T1**	0.37 (0.11) **a**	0.36 (0.12) **a**	0.16 (0.13) **b**	0.18 (0.11) **b**	<0.001 *
	**T3-T2**	0.05 (0.08)	0.05 (0.07)	0.01 (0.13)	0.02 (0.13)	0.62
**1.5 mm**	**T2-T1**	0.17 (0.10)	0.19 (0.05)	0.21 (0.08)	0.21 (0.09)	0.74
	**T3-T1**	0.20 (0.10)	0.22 (0.06)	0.25 (0.05)	0.26 (0.06)	0.31
	**T3-T2**	0.02 (0.09)	0.03 (0.10)	0.04 (0.11)	0.05 (0.11)	0.92

LD: IPS Emax, ZLS: Celtra Duo, ALD: Cerec Tessera, LE: IPS Empress. T2-T1: Difference between results after toothbrushing (without toothpaste) and before toothbrushing. T3-T1: Difference between results after toothbrushing (with toothpaste) and before toothbrushing. T3-T2: Difference between results after toothbrushing (with toothpaste) and toothbrushing (without toothpaste). The Kruskal–Wallis test was used. * Statistically significant at *p* value < 0.05. **a**, **b**: Different lowercase letters denote statistically significant differences between the four groups using the Bonferroni-adjusted significance level.

**Table 3:** Microhardness of the four study groups at different thicknesses.

Thickness	LD (n = 10)	ZLS (n = 10)	ALD (n = 10)	LE (n = 10)	*p* Value
	Mean (SD)				
**0.5 mm**	879.15 (29.57) **a**	949.42 (37.96) **b**, **A**	973.37 (67.29) **b, A**	880.27 (40.61) **a**	<0.001 *
**1 mm**	888.82 (29.70) **a**	978.05 (37.60) **b**, **AB**	1016.75 (42.77) **b**, **AB**	886.48 (58.99) **a**	<0.001 *
**1.5 mm**	900.18 (48.85) **a**	1005.28 (34.43) **b**, **B**	1066.92 (69.54) **b, B**	896.63 (41.13) **a**	<0.001 *
***p*** **value**	0.46	0.008 *	0.008 *	0.74	

LD: IPS Emax, ZLS: Celtra Duo, ALD: Cerec Tessera, LE: IPS Empress. * Statistically significant at *p* value < 0.05. **a**, **b**: Different lowercase letters denote statistically significant differences between the four groups using the Bonferroni-adjusted significance level. **A**, **B**: Different uppercase letters denote statistically significant differences between the different thicknesses in the same group using the Bonferroni-adjusted significance level.

**Table 4:** Three-way ANOVA assessing the associations of the different factors with the surface roughness (Ra) in µm.

		Adjusted Mean (SE)	95% CI	*p* Value
**Group**	**LD**	0.77 (0.007) **a**	0.76, 0.79	<0.001 *
	**ZLS**	0.91 (0.007) **b**	0.90, 0.93	
	**ALD**	0.64 (0.007) **c**	0.63, 0.66	
	**LE**	0.73 (0.007) **d**	0.72, 0.75	
**Thickness**	**0.5 mm**	0.73 (0.006) **a**	780.48, 811.34	<0.001 *
	**1 mm**	0.77 (0.006) **b**	777.55, 808.41	
	**1.5 mm**	0.80 (0.006) **c**	805.56, 836.42	
**Toothbrushing**	**T0**	0.61 (0.006) **a**	664.95, 695.81	<0.001 *
	**T1**	0.83 (0.006) **b**	850.13, 880.99	
	**T2**	0.86 (0.006) **c**	848.51, 879.36	

Model F: 227.004, *p* value < 0.001. Adjusted R^2^: 0.82. LD: IPS Emax, ZLS: Celtra Duo, ALD: Cerec Tessera, LE: IPS Empress. SE: Standard error, CI: Confidence Interval. T1: Before toothbrushing. T2: After toothbrushing (without toothpaste). T3: After toothbrushing (with toothpaste). * Statistically significant at *p* value < 0.05. **a**, **b**, **c**: Different letters denote statistically significant differences between groups using the Bonferroni-adjusted significance level.

**Table 5:** Two-way ANOVA assessing the associations of different factors with the hardness in mm.

		Adjusted Mean (SE)	95% CI	*p* Value
**Group**	**LD**	889.38 (8.64) **a**	872.27, 906.49	<0.001 *
	**ZLS**	977.58 (8.64) **b**	960.47, 994.69	
	**ALD**	1019.01 (8.64) **c**	1001.90, 1036.12	
	**LE**	887.79 (8.64) **a**	870.68, 904.90	
**Thickness**	**0.5 mm**	920.55 (7.48) **a**	905.74, 935.37	<0.001 *
	**1 mm**	942.53 (7.48) **ab**	927.71, 957.34	
	**1.5 mm**	967.25 (7.48) **b**	952.43, 982.07	

Model F: 38.48, *p* value < 0.001. Adjusted R^2^: 0.61. LD: IPS Emax, ZLS: Celtra Duo, ALD: Cerec Tessera, LE: IPS Empress. SE: Standard error, CI: Confidence Interval. T1: Before toothbrushing. T2: After toothbrushing (without toothpaste). T3: After toothbrushing (with toothpaste). * Statistically significant at *p* value < 0.05. **a**, **b**, **c**: Different letters denote statistically significant differences between groups using the Bonferroni-adjusted significance level.

## Data Availability

Data supporting the reported results can be requested from the corresponding author.
